# Evaluation of Left Ventricular Endocardial Cardiac Resynchronization Therapy in a Non-responder with Ventricular Arrhythmias

**DOI:** 10.1016/s0972-6292(16)30713-6

**Published:** 2014-01-01

**Authors:** Masih Mafi Rad, Yuri Blaauw, Luuk Debie, Hans-Peter Brunner-La Rocca, Kevin Vernooy

**Affiliations:** Maastricht University Medical Center, Department of Cardiology, Post office box 5800, 6202 AZ Maastricht, the Netherlands

**Keywords:** Cardiac resynchronization therapy, Ventricular arrhythmia, Acute invasive hemodynamic measurements

## Abstract

Approximately one third of patients treated with cardiac resynchronization therapy do not derive any detectable benefit. In these patients, acute invasive hemodynamic evaluation can be used for therapy optimization. This report describes the use of systematic invasive hemodynamic measurements for clinical decision making in a patient who experienced severe ventricular arrhythmias and clinical deterioration following a biventricular upgrade.

## Introduction

Cardiac resynchronization therapy (CRT) has been established as an effective treatment for heart failure patients with severe left ventricular (LV) systolic impairment and electrical dyssynchrony [1]. Unfortunately, a significant proportion of apparently suitable patients fail to benefit [2]. In this subset of patients, acute invasive hemodynamic evaluation can be used to optimize CRT with alternative LV pacing sites or atrio-ventricular (AV) and inter-ventricular pacing delays [3,4]. We describe a patient who experienced severe ventricular arrhythmias and clinical deterioration following upgrade to a biventricular (BiV) system. CRT was discontinued, based on the outcome of invasive hemodynamic measurements.

## Case Report

**Figure 1:**
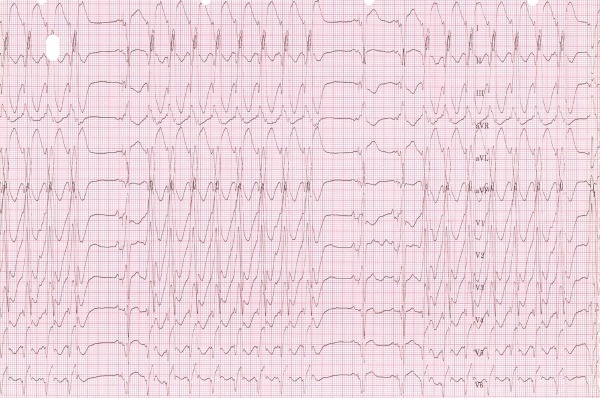
Incessant VT, 4 weeks after onset of BiV pacing

A 65-year-old male patient with non-ischemic dilated cardiomyopathy, left ventricular ejection fraction of 20%, intra-ventricular conduction delay with a QRS duration of 148 ms, and NYHA class 3 was scheduled for upgrade of an implantable cardioverter defibrillator (ICD) to a cardiac resynchronization therapy - defibrillation (CRT-D) system. The ICD had previously been implanted for primary prevention. Echocardiography prior to the BiV upgrade demonstrated myocardial scarring of the LV lateral wall and showed no evidence of mechanical dyssynchrony. Unfavorable coronary venous anatomy required epicardial LV lead placement in the lateral position via minimal lateral thoracotomy. Four weeks later, the patient was admitted with multiple ICD discharges due to incessant ventricular tachycardia (VT) (Figure 1). He was sedated and Amiodarone was initiated, whereupon VT recurrences were no longer observed. In the following months his clinical condition deteriorated despite continuation of BiV pacing.

**Figure 2:**
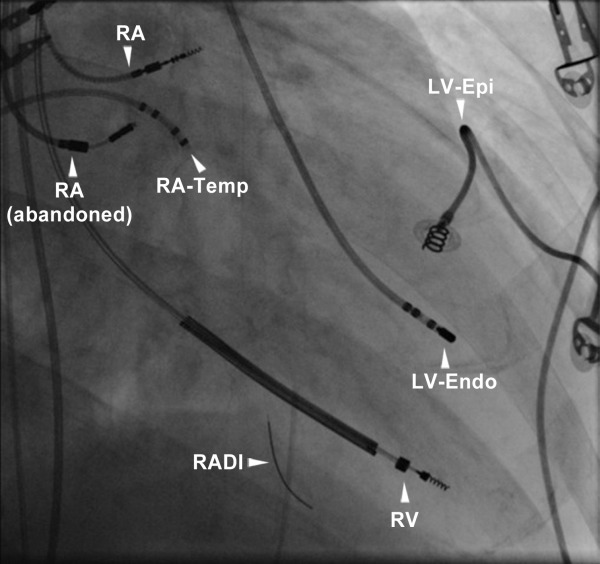
X-ray detail of the invasive hemodynamic pacing study setup. The right atrial (RA), right ventricular (RV) and LV epicardial (LV-Epi) lead of the implanted system are shown, together with the temporary right atrial (RA-Temp) and LV endocardial electrode (LV-Endo). The RADI pressure wire is located in the LV cavity

An invasive hemodynamic pacing study was performed to evaluate the hemodynamic effect of conventional BiV pacing and to explore whether LV endocardial pacing could effectuate hemodynamic improvement. For this purpose, a temporary pacing electrode and a RADI pressure wire were positioned within the LV cavity, another temporary pacing electrode was placed in the right atrium (Figure 2). The RADI wire allowed determination of maximal rate of LV pressure rise (LVdP/dtmax ) as a measure of LV function. The pacing protocol consisted of a baseline without ventricular pacing (atrial pacing) alternated with AV-sequential BiV, LV and right ventricular (RV) pacing using the implanted system and LV endocardial pacing using the temporary LV pacing electrode. To exclude hemodynamic variability induced by spontaneous changes in heart rate, pacing was performed at a faster rate 10 beats above intrinsic heart rate. Average LVdP/dtmax was measured during 10 seconds of pacing for each pacing configuration.

**Figure 3:**
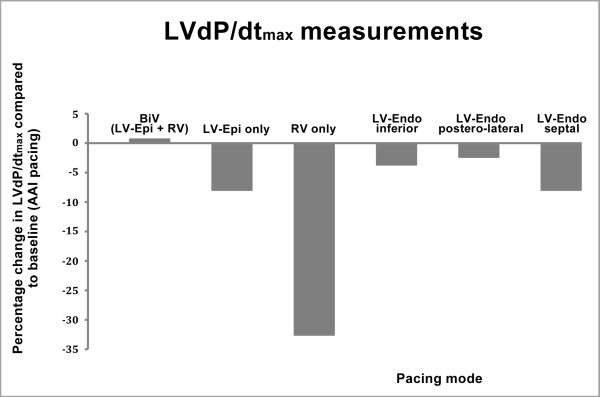
Percentage change in LVdP/dtmax during AV-sequential BiV, LV and RV only pacing using the implanted system and LV endocardial pacing at different sites using the temporary LV endocardial electrode compared to baseline (AAI pacing). Epi = Epicardial, Endo = Endocardial

**Figure 4:**
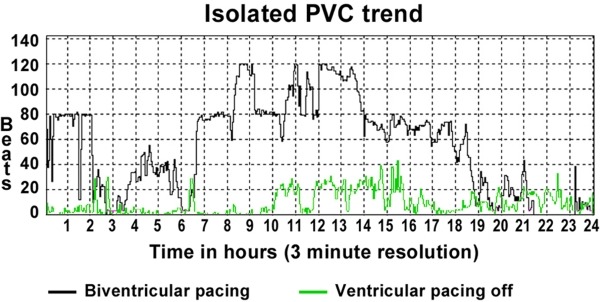
24 hour Holter recordings demonstrating 40,000 PVCs during BiV pacing versus 4000 PVCs during ventricular pacing programmed off (AAI mode)

Neither LV, RV or BiV pacing with the implanted system nor LV endocardial pacing at different sites improved LVdP/dtmax as compared to baseline (Figure 3). Furthermore, the number of premature ventricular complexes (PVCs) increased upon LV stimulation. This was confirmed by 24 hour Holter monitoring, which demonstrated 40,000 PVCs during BiV pacing versus 4000 PVCs when ventricular pacing was programmed off (Figure 4). Considering the unfavourable hemodynamic and pro-arrhythmic effects of LV pacing, placement of a permanent endocardial LV lead was not performed and the ICD was programmed to ventricular pacing off (AAI-DDD mode). Our patient was scheduled for left ventricular assist device placement.

## Discussion

Patient selection plays an important role in CRT response. According to the ESC heart failure guidelines available at the time of device implantation, our patient had a class I indication for CRT [5]. However, based on the most recent guidelines, he may not have been an "ideal" candidate due to the absence of a typical left bundle-branch block [12]. This is supported by the outcome of the acute hemodynamic measurements. Although a definite relationship between acute LVdP/dtmax increase and long term clinical response needs to be confirmed, our findings suggest that acute invasive hemodynamic testing could help to guide the decision on whether or not to implant a CRT device in cases where there is less consensus for a clinical benefit.

The presence of myocardial scar at the area targeted by the LV lead is an important determinant of CRT response [6]. Lead position optimization in the coronary sinus is usually limited by side branch anatomy. A surgical epicardial LV lead implantation may then be preferred. Our patient had extensive scarring of the LV lateral wall which limited the possibility of placing an epicardial LV lead outside the scar region. Recently, LV endocardial pacing has been suggested as a potentially better alternative to the epicardial approach [7]. This method allows more liberty in lead positioning, which can help to avoid pacing in infarcted areas. However, the risk of thrombo-embolic complications is unknown and requires further investigation. Although the potential of acute LVdP/dtmax measurements to predict long-term outcome needs to be confirmed, our findings suggest that acute hemodynamic testing of LV endocardial pacing in a temporary setup may help to identify the optimal pacing site and may also prevent implantation of a potentially profitless endocardial LV lead with a yet unknown risk of hazardous complications.

In a small percentage of patients, CRT may potentiate ventricular tachyarrhythmias. The exact mechanisms of proarrhythmia remain largely unclear but our findings suggest that pacing in the region of myocardial scar may facilitate its development. This conception is supported by the morphology of the VT which suggested a lateral exit site close to the LV epicardial pacing lead. A suggested underlying mechanism is a potential increase in local myocardial oxygen demand by pacing, precipitating ischemia in the peri-infarct zone and making it vulnerable to re-entry [8,9]. The currently available evidence is however too limited to develop strategies for stratifying patients at risk for proarrhythmia in the setting of CRT.

## Conclusion

This case report illustrates the potential contribution of acute invasive hemodynamic evaluation to clinical decision making in CRT. Acute hemodynamic testing may also be considered as a step-up approach to justify implantation of a LV endocardial lead and to guide lead positioning optimization. Finally, this case underlines the importance of considering the possibility of CRT related proarrhythmia, whenever CRT treated patients develop new ventricular tachyarrhythmia's.
